# Changing Trends of Consumers' Online Buying Behavior During COVID-19 Pandemic With Moderating Role of Payment Mode and Gender

**DOI:** 10.3389/fpsyg.2022.919334

**Published:** 2022-08-10

**Authors:** Sana Sajid, Rao Muhammad Rashid, Waleej Haider

**Affiliations:** ^1^Management Studies Department, Bahria University, Karachi, Pakistan; ^2^Faculty of Engineering Sciences and Technology, Hamdard University, Karachi, Pakistan

**Keywords:** perceived benefits, perceived ease of use, perceived enjoyment, social influence, behavioral intention, actual behavior, gender, payment mode

## Abstract

It was not long ago when technological emergence fundamentally changed the landscape of global businesses. Following that, business operations started shifting away from traditional to advance digitalized processes. These digitalized processes gave a further boost to the e-commerce industry, making the online environment more competitive. Despite the growing trend, there has always been a consumer market that is not involved in online shopping, and this gap is huge when it comes to consumers from developing countries, specifically Pakistan. On contrary, the recent COVID-19 pandemic has brought drastic changes to the way consumers used to form their intention and behave toward digitalized solutions in pre COVID-19 times. Evidence shows that the global e-commerce industry has touched phenomenal growth during COVID-19, whereas Pakistan's e-commerce industry still holds a huge potential and has not fully boomed yet. These facts pave new avenues for marketers to cater to this consumer market for long-term growth. Hence, the study provides insights into how consumers' online buying behavior has transformed during the COVID-19 pandemic in the context of Pakistan. The study presents a framework based on the Technology Acceptance Model (TAM) and Theory of Planned Behavior (TPB). Furthermore, the moderating role of gender and payment mode has also been examined. For the analysis of variables, the partial least squares (PLS) method was used to conduct structural equation modeling (SEM) by collecting data from 266 respondents. The results show a significant and positive impact of perceived benefits, perceived ease of use, perceived enjoyment, and social influence on consumers' intention, but they also show an insignificant impact of gender and payment mode as a moderating variable on PEOU-BI and BI-AB, respectively. The results are of utmost significance for Pakistani businesses, marketers, and e-traders to streamline their business practices accordingly. Lastly, the proposed framework demonstrates new directions for future research to work upon.

## Introduction

In developing countries like Pakistan, the Internet brought much convenience to businesses, specifically in the twenty-first century. Due to the current COVID 19 pandemic, there has been a drastic change in the way consumers have shifted toward online buying. It is evident that post-COVID circumstances have left a significant impact on the e-commerce industry (Rashid et al., 2022). This has caused global e-commerce sales projection to reach $7.4 trillion by 2025 (Statista, 2022). On the contrary, South Asian countries show a low share of just 1.4% in global e-commerce business compared to their population share in the world.

Pakistan's e-business has shown drastic improvements ever since the pandemic struck. As per SBP (FY20), registered e-commerce merchants have increased, and markets have expanded to Rs. 234.6 billion with 55.5% yearly. These situations have raised doubts about Pakistan's digital connectivity, which shows a huge potential for growth and untapped areas for e-traders in Pakistan. Despite these statistics, the e-commerce market in Pakistan is still in its infancy stage. It has been evident that there are a whole lot of consumer bases that are not involved in online shopping (Ahmed et al., 2017). The reason for this lack of involvement in online shopping is unknown. Domestically, there is a research and development gap that causes inconsistency in theoretical and empirical evidence on factors that may shape an individual's online buying behavior in the Pakistani market. Therefore, it is pertinent to fill this gap by examining Pakistani consumers' psychological and behavioral beliefs. Globally, there has been plenty of research studies conducted proposing valuable conceptually, theoretically, and empirically tested frameworks that intend to explain antecedents of consumers' intentions toward online buying behavior.

These studies examined the online behavior of consumers in numerous dimensions and postulated perceptions behind online shopping behavior and attributes (Jarvenpaa and Todd, 1996; Chang and Kannan, 2006), consumer information process styles, online store layouts, (Park and Kim, 2003), behavioral and normative beliefs about technology adoption (Karahanna et al., 1999; Limayem et al., 2001; Foucault and Scheufele, 2002) risks related to online shopping (Jarvenpaa et al., 1999; Akhlaq and Ahmed, 2015; Haider and Nasir, 2016; Pappas, 2016), and technology-oriented factors affecting online purchase intention (van der Heijden et al., 2003; Prashar et al., 2015).

Overall, the connotations of previous studies tended toward two dimensions: (a) “product and shopping attributes” that are customer-specific and (b) “technological attributes” that are website-/technology-specific. None of the available studies has covered integrated attributes of both dimensions “customer-specific” and “technology-specific” in a single framework to study insights of consumers' behaviors for online buying. Hence, there is a need to address underlying factors that may shape consumers' intention and actual behavior to opt for online purchases. Based on these arguments, the present study proposes a comprehensive framework comprised of factors impacting consumers' online buying behavior during the pandemic.

In this regard, the theoretical foundation of this study is built upon the Technology Acceptance Model (Davis, 1989) (TAM), which is an extension of the Theory of Planned Behavior (TPB) (Ajzen, 1985). TAM is a widely used and highly influential model of user's acceptance of “technology.” As the present study examines the buying behavior of an online consumer, it tends to predict how consumers' perceived benefits, perceived ease of use, perceived enjoyment, and social influence have an impact to form consumers' intention and behavior to purchase online.

The results of this study would be of interest to a diverse research audience, including the academia, marketers, advertisers, policymakers, governments, and businesses. For the academia, new theoretical literature has been presented with the inclusion of potent constructs obtained from the technological model (TAM), psychological, and behavioral model (TPB) along with the normative notion of social influence on consumers' behavioral intention and behavior. Marketers may devise strategies to encourage their consumers to opt for online purchases, whereas advertisers may use appealing and creative content to promote them. In addition, policymakers may enact laws to encourage e-trading, and the government may facilitate Pakistani e-traders by releasing funds to build and maintain an advanced IT infrastructure. Lastly, the study would be of optimum significance for businesses that may work on their website designs and processes and maintain a website infrastructure to aid consumers according to their changing shopping preferences.

## Literature Review

### Perceived Benefits and Behavioral Intentions

Previous studies have provided many findings and devoted considerably to delivering benefits to consumers to stimulate their shopping intentions. Research has clearly defined the concept of consumer benefits and the significance of hedonic and utilitarian benefits for them (Babin et al., 1994; Holbrook, 1994; Jones et al., 2006; Wang et al., 2013). Consumers derive practical benefits from the performance of a product or a service after achieving a task (Kim, 2002). Furthermore, recent studies conducted by Widyastuti et al. (2020) stated the perception of perceived benefits, whereas Yew and Kamarulzaman (2020) and Bangkit et al. (2022) found a significant positive impact of perceived benefits on online consumer behavior. In the same line, a study conducted by Jeong et al. (2003) on “online shoppers of the hotel industry” found that for customers, the most critical factor that influences their “behavior intention” is the satisfaction level of available information, dimensions, and attributes provided by a website. Chang and Kannan (2006) stated in their study that website quality has positively influenced consumers' purchase intention. Bai et al. (2008) found significantly positive empirical results in online usability, functionality, customer satisfaction, and behavior intentions. The study further stated that consumers perceive all these dimensions as valued, increasing their purchase intentions. As Babin and Babin (2001) stated that consumers who efficiently complete shopping tasks would show stronger repeated purchase intentions.

In addition, Teo (2002), Xia et al. (2008), Nazir et al. (2012), and Manu and Fuad (2022) shared similar findings where consumers derive attributes of perceived benefits through online shopping; it provides the required information on a product or a service, saves time, low prices, and convenience in the availability of products that are not locally available. Online shopping is getting popular in Pakistan because of its ease of use and the comfort it brings to consumers without much effort (Iqbal and Hunjra, 2012). Furthermore, research highlights that consumers seek internet shopping valuable for price reviews and comparisons, search and deal evaluation convenience, low prices, selection variety, information on product features, latest awareness of brands and fashion trends (Sorce et al., 2005; Zhou and Zhang, 2007; Jiang et al., 2013; Jhamb and Gupta, 2016). Teo (2006) indicates that consumers expect benefits like sufficient product information, convenience, online security, and easy contact with vendors. Moreover, while shopping online, consumers also expect prompt delivery of a product, a reliable supply chain, and return transaction policies (Dawn and Kar, 2011).

H1: Perceived benefits significantly impact the behavioral intention for online purchases.

### Moderating Role of Gender

In various marketing and consumer behaviors, demographic variables, specifically the impact of gender, have been taken into different contexts. In some studies, overall demographics are used as antecedents of TAM variables (Porter and Donthu, 2006). Others have used them to moderate the effect of the predictor and criterion relationship in technological acceptance (Chang and Kannan, 2006). Previously, research studies have accepted that there is a significant role of gender in technology acceptance (Yousafzai and Yani-de-Soriano, 2012); a study further shows that men have a more strong and significant impact on perceived usefulness and behavioral intention in relation to technology acceptance and women have more impact on perceived ease of use and behavioral intention. This study is in line with Davis (1989), Clegg and Trayhurn (2000), and Venkatesh et al. (2003). In conclusion, it has been argued that men are more tech-savvy, task-oriented and adopt technology to avail themselves benefits of online shopping. However, for acceptance of technology, women tend to show more computer anxiety than men (Venkatesh and Morris, 2000; Karavidas et al., 2005; Zhang, 2005).

H2: Gender moderates the effect of perceived benefits on the behavioral intention for online purchase.
H3: Gender moderates the effect of perceived ease of use on the behavioral intention for online purchase.

### Perceived Ease of Use (PEOU) and Behavioral Intention (BI)

Perceived ease of use is best defined by Davis (1989, 1993) as one of TAM's basic constructs. PEOU is defined as a degree to which a person believes using a particular system is effortless (Davis, 1989). Al-Azzam and Fattah (2014) postulated that perceived ease of use refers to a consumer who believes that using the Internet for shopping is free of effort and involves minimal friction in using and handling websites. Apart from the vital role of “ease of use” in technology acceptance, it has also been proposed for website usability and efficiency while shopping online (Monsuwe et al., 2004). Considering these findings, it can be claimed that if there is an ease in usage and effortlessness in handling technology, consumers are more likely to adopt a system while purchasing online. Hence, one's intention to purchase online increases (Venkatesh, 2000; Xia et al., 2008). Many other researchers have confirmed a strong sign and a direct relationship between perceived ease of use and the behavioral intention of a person (Teo et al., 1999; Venkatesh and Bala, 2008; Ingham et al., 2015).

The study further implies that if a consumer has an increased experience, they adjust themselves to system-specific ease of use and reflect on their interaction with repeated usage of the system, which influences the behavioral intention to shop online. Few latent dimensions merely shape “ease of use” including site characteristics, navigation, and download speed (Zeithmal et al., 2002). However, the most significant role in shaping “ease of use” is played by two dimensions elaborated by Venkatesh (2000); these include computer self-efficacy, computer anxiety, and computer playfulness; “computer self-efficacy” relates to the general use of computer or skills needed to operate a system; “computer anxiety” refers to a person's fear of using a computer when required, whereas “computer playfulness” is a degree to which a consumer's cognitive ability makes them feel less effortful and underestimates the complexity of system usage for online interaction. Increased usage experience contributes to unique attributes of perceived enjoyment concerning user system specification; it makes a more enjoyable experience for users. (Venkatesh, 2000; Monsuwe et al., 2004).

H4: PEOU significantly impacts behavioral intention for online purchase.

### Perceived Enjoyment (PE) and Behavioral Intention (BI)

Researchers have explained enjoyment as how online shopping is perceived to be enjoyable or fun for a consumer. Various researchers have theoretically and empirically proved the role of intrinsic motivation in online shopping (Davis et al., 1992; Venkatesh and Speier, 1999; Venkatesh, xbib2000). Intrinsic motivation has been taken as a construct of perceived enjoyment in many studies (Monsuwe et al., 2004). Davis et al. (1992) introduced the third belief in TAM, perceived enjoyment. He proposed that perceived enjoyment directly impacts the behavioral intention of an online consumer. In addition, studies conducted in the past two decades have shed some light to state the role of perceived enjoyment in the behavioral intention of a consumer (Koufaris, 2002; Cyr et al., 2006; Chang and Chen, 2008; Marza et al., 2019; Bangkit et al., 2022). Triandis (1980) reports that emotions like fun, joy, and pleasure influences human behavior. According to self-determination theory (Deci, 1975), if a person is intrinsically involved in online shopping and personally determined, they enjoy doing it. Kuswanto et al. (2019) investigated variables impacting the online behavior of university students in Indonesia and highlighted that the online shopping behavior of consumers significantly gets influenced by enjoyment, social influence, and perceived risk.

Furthermore, a study conducted by Akhlaq and Ahmed (2015) has also proposed perceived enjoyment as a significant construct backed by an intrinsic motivation that positively impacts consumers' intention to shop online. Findings on Pakistani consumers reported by Cheema et al. (2013) show that perceived enjoyment has a significant and positive impact on online shopping intention and holds a 42% contribution to the model. Apart from intrinsic motivations, another latent dimension, exploration and curiosity to use a system, is also prominent in investigating the online shopping context. The empirical evidence reported by Teo (2002) shows that interest in online browsing is related to curiosity about knowing various products and brands available to purchase online. According to Teo's study, around 50% of the respondents browsed even if they did not intend to purchase.

H5: Perceived enjoyment mediates the relationship between perceived ease of use and behavioral intention for online purchase.

### Causal Nature of Perceived Ease of Use (PEOU) and Perceived Enjoyment (PE)

There are differences in research findings that confirm the causal relationship between perceived ease of use and perceived enjoyment (Sun and Zhang, 2006a,b). In some studies, perceived enjoyment has been considered as an antecedent of perceived ease of use (Venkatesh, 1999, 2000; Agarwal and Karahanna, 2000; Venkatesh et al., 2002). In other studies, it has been confirmed as a consequence of perceived ease of use (Deci, 1975; Davis et al., 1992; Teo et al., 1999; van der Heijden et al., 2003). It has been claimed that an easier-to-use system is more enjoyable (Igbaria et al., 1995). For an empirical discussion of this inconsistent argument regarding the causal relationship between perceived ease of use and enjoyment, Sun and Zhang (2006a,b) conducted information system-based research in a utilitarian context using a covariance-based statistical method to find a causal relationship. They concluded that perceived enjoyment and perceived ease of use have overall dominance in the model in a utilitarian system environment. The present study aims to confirm this causal relationship by considering perceived enjoyment due to perceived ease of use. The study tends to measure a consumer's buying behavior *via* technology (Davis et al., 1992; van der Heijden et al., 2003).

H6: Perceived ease of use significantly impacts perceived enjoyment for online purchase.

### Social Influence (SI) and Behavioral Intention (BI)

“Social influence” (SI), an antecedent of the subjective norm (SN), is a crucial construct of TPB and TAM (Davis, 1989) that has originated from the Theory of Reasoned Action (TRA) (Fishbein and Ajzen, 1975). TRA states that a person's behavioral intention (BI) has a significant and positive relationship with subjective norms (Karahanna et al., 1999). One's social circle may influence a person to behave in a particular manner (Ajzen, 1985). According to classic internalization studies, when someone incorporates the referent's influence in adopting a system, the person perceives the referent's belief as their own belief (Kelman, 1958; Warshaw, 1980). Wei et al. (2009) mentioned in their study Rogers (1995)' proposition of social influence; he stated that social influence can be defined as two forms: mass media and interpersonal influence. Mass media or external influence includes newspapers, reports, academic journals, published articles, magazines, television, radio, and other applicable mediums, whereas interpersonal influence comes from family, peers, friends, social networks, and electronic word of mouth (EWOM) (Bhattacherjee, 2000; LaRose and Eastin, 2002; Rao and Troshani, 2007; Pietro et al., 2012).

Venkatesh and Davis (2000) stated that people incorporate social influence to gain status and acceptance in their social setting. Studies by Ketabi et al. (2014) and Kuswanto et al. (2019) further highlighted the role of social norms and social influence on consumers, respectively; it has been stated that in certain situations the reference group of a person, specifically “friends,” strongly influences the behavior of an individual. In their qualitative study, Wani et al. (2016) also identified “social influence” and “e-word of mouth” as critical factors. Their study further elaborates that the opinions of consumers, peers, friends, and colleagues matter a lot while purchasing online. Even in the last shopping stage, just before check-out, if a consumer reads any comment about a product or a service, it undoubtedly impacts one's decision (Park et al., 2011).

H7: Social influence significantly impacts behavioral intention for online purchase.

### Behavioral Intention (BI) and Actual Behavior (AB)

Plenty of studies have used TPB and TAM to determine an individual's intention to engage in a particular behavior (Ajzen, 1985; Pavlou and Marshall, 2002; Delafrooz et al., 2010; Tsai et al., 2011; Zaidi et al., 2014; Bauerová and Klepek, 2018). Behavioral intention is the focal point of TRA, TPB, and TAM. According to the extended TAM model postulated by Venkatesh and Davis (2000), the relationship between intention to use and actual usage was significant and strongly mediated the effect of perceived benefits, perceived ease of use, and subjective norms on actual usage of an online consumer. Limayem and Hirt (2003) elaborated in their study that there are some “facilitating conditions” (Triandis, 1979) that moderate the relationship between behavioral intention and actual behavior. Even if a person intends to act in a certain way, one cannot without those facilitating conditions. These conditions align with Ajzen's “perceived behavioral conditions,” but the point of difference is that Ajzen's perceived behavioral control is subjective, whereas Triandis' facilitating conditions are objective. In the present study, the behavioral intention was considered to examine the significance of perceived intentions of a person on actual buying behavior while purchasing online.

### Payment Mode

According to a report by Yousuf (2018) [Asian Development Bank (ADB)], 95% of Pakistan's e-commerce transactions come from cash on delivery (COD), and the remaining 5% comes from electronic payment with debit/credit cards. Another study states that only 31% of Pakistani tend to pay online for shopping and that cybercrimes and lack of trust in payment systems are the main reasons for their choice. (CIGI, 2017). An increase in the online payment rate includes uncertain security and privacy issues that may influence consumers' buying behavior in e-markets (Pang et al., 2016). As Valois et al. (1988) stated, some factors may affect the strength of the relationship between intention and actual behavior. However, in the current perspective of the COVID-19 pandemic, it has been stated by Pollak et al. (2022) that the market has become adaptable to non-standard situations within a short period. This implies that there have been specific changes observed in consumers' behaviors and preferences during crisis times. Considering the facts and figures on the “payment mode” of Pakistan's e-commerce and the overall concept of “facilitating conditions” from Triandis' theory, the study tends to imply that the “online payment method” (OPM) is a moderator to determine the impact of payment method on the relationship between intentions and actual behavior.

H8: Payment mode moderates the effect of behavioral intention on actual behavior for online purchase.
H9: Behavioral intention mediates the impact of perceived benefits, perceived ease of use, social influence, and perceived enjoyment on actual behavior for online purchase.

**Figure 1:**
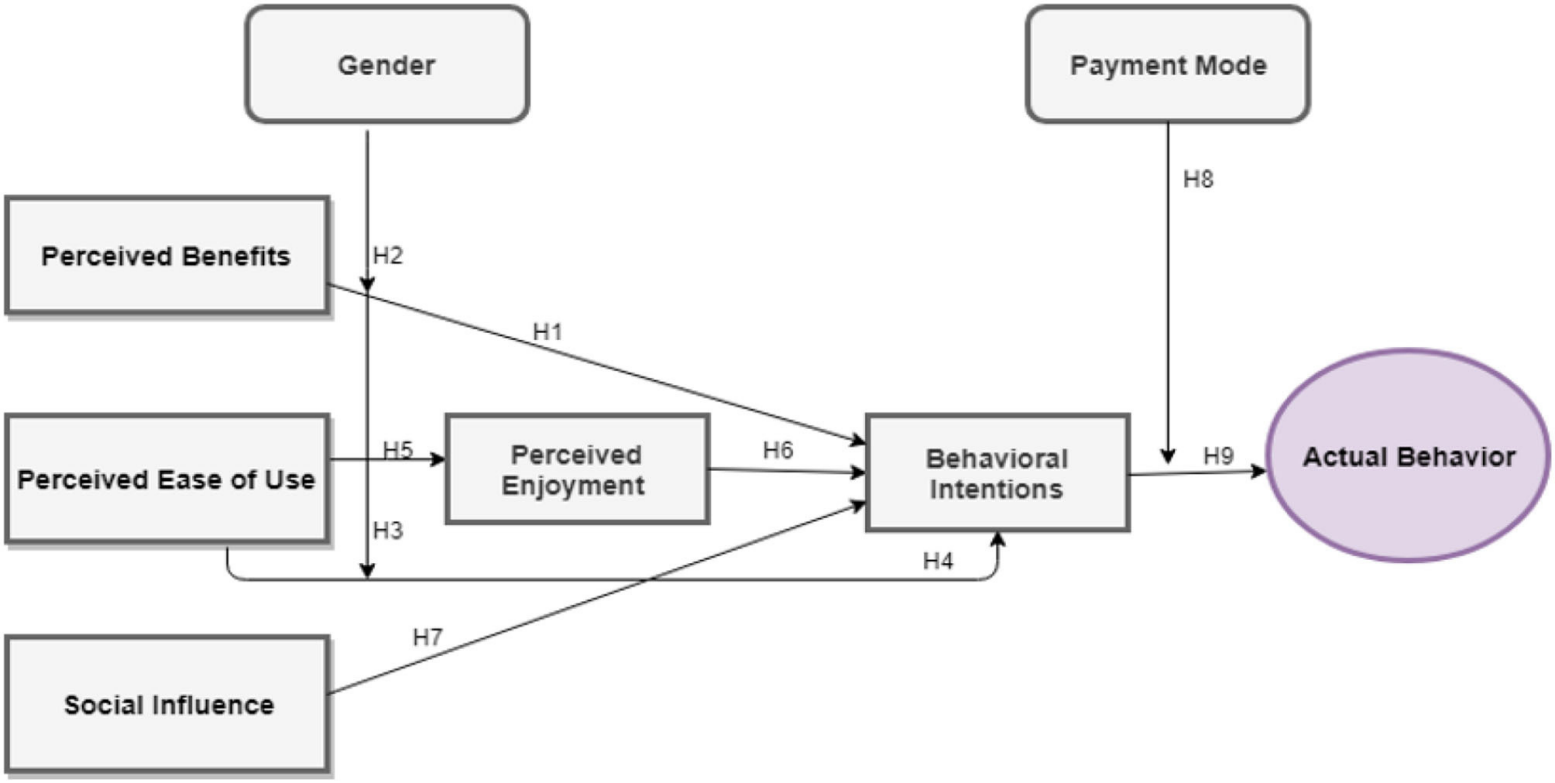
Theoretical framework.

Based on hypotheses this study builds the framework to study four variables namely, perceived benefits, perceived ease of use, perceived enjoyment, and social influence along with mediating role of behavioral intention, whereas moderating role of Gender and payment mode is also under examination for the study (Figure 1).

## Methodology

### Instrument Development

This study conducted an online survey for data collection. In this regard, an adapted instrument from previous studies was used and tested for reliability and validation. Questionnaires were sent to respondents for collection of their responses on a five-point Likert scale, which ranged from 1 (Strongly Disagree) to 5 (Strongly Agree). It is in line with previous studies conducted in the context of online consumer buying behavior (Davis et al., 1992; van der Heijden et al., 2003; Sorce et al., 2005; Yang et al., 2015).

Furthermore, for unit analysis, eight items of perceived benefits were adapted from Teo (2002), Swinyard and Smith (2003), Sorce et al. (2005), and Forsythe et al. (2006). Five items of perceived ease of use were adapted from Gefen et al. (2003) and Cheema et al. (2013). Four items of perceived enjoyment were adapted from Teo (2002) and Cheema et al. (2013). Five items of social influence were adapted from Davis (1985) and Karaiskos et al. (2012). Three items of behavior intention were adapted from Limayem and Hirt (2003)) and Karaiskos et al. (2012). In addition, three items of actual behavior were adapted from Karaiskos et al. (2012). Lastly, the items of payment mode were adapted from Hasan and Gupta (2020).

Furthermore, the questionnaires contain two sections; the first section contains demographic variables of an individual including age, gender, and education level, whereas the second section contains the independent, moderating, and mediating variables of the study.

### Sample and Procedures

For data collection of the present study, 350 questionnaires were sent out to respondents who were online buyers. The questionnaires were developed with questions regarding whether respondents are online buyers, how long they have been into online buying, and what is the occurrence of their buying patterns. In addition, the questionnaire link shared with the respondents included a note stating that this study seeks responses from online buyers only and that respondents who were not online buyers were not required to record responses.

A self-administered questionnaire was sent out using “an online survey”. A questionnaire link was sent out to the respondents *via* social media platforms and email. Out of 350, a total of 266 responses were received, and no data were missing from the 266 responses as the questionnaires were designed by utilizing close-ended questions to choose from the list, and fields were marked required. According to the gender category, of those who participated in the research, 51.5% were men and 48.5% were women. The remaining demographic details are shown in Table 1.

**Table 1:** Respondents' profile.

**Measures**		**Frequency**	**%**
Gender	Male Female	137 129	51.5 48.5
Age	15–25 26–35 36–45 45 and above	93 157 10 6	35 59 3.8 2.3
Educational level	Intermediate Bachelors Masters Postgraduate	17 98 103 36	6.4 36.8 38.7 13.5
Online shopper since	1–2 year 3–4 year More than 5 years	143 67 56	53.8 25.2 21.1
Frequency of online shopping	Frequent Online shopper Sometimes Shop online Occasionally Online shopper	108 73 85	40.6 27.4 32

### Evaluation Method

The partial least squares (PLS) method was used to conduct the structural equation modeling (SEM) approach to evaluate the present study. Hair et al. (2012) stated that PLS is a second-generation evaluation technique that measures and tests structural modeling, component factor analysis (CFA), and regression. Thus, extensive pre-analysis and data validation were conducted using Smart-PLS for the present study.

## Result Analysis

### Common Method Bias

Kock (2015) stated that the occurrence of variance inflator factor (VIF) should be less than or equal to 3.3. For this study, all VIF values were in the range of 1.67- 2.6, showing that the model was considered free of common method bias because no such thing was observed. To further testify the model, Harman's single-factor test (Podsakoff and Lee, 2003) was conducted to examine if the model was free from common method biases. According to the requirement, if the total variance extracted by one factor exceeds 50%, this shows the presence of common method biases in the study. However, the present study shows that the total variance extracted by one aspect is 27.288, less than 50%. Also, the inter-correlation of all the constructs of this study is less than 0.9 (Pavlou and El Sawy, 2006). Hence, the outcomes indicate that common method bias is not an issue in this study.

### Measurement Model

An assessment of reliability and validity was conducted to evaluate and reduce measurement errors. It has been stated as a required test to reduce measurement errors while assessing for internal consistency, discriminant, and convergent validities (Hair et al., 2012). Furthermore, these tests have been evaluated by assessing the values of Cronbach's alpha (α), factor loadings, average variance extracted (AVE), and composite reliability (CR). The acceptable value of CFA should be 0.7 at minimum (Hair et al., 2012). Along similar lines, the present study shows that the CFA values are above 0.7 and are acceptable to show the internal consistency of the data (Table 2). Furthermore, for all the constructs, the values of AVE and CR are above 0.5 and 0.8, respectively (Fornell and Larcker, 1981); these values show acceptable convergent validity. Table 2 shows that Cronbach's alpha, CR, and AVE of actual behavior are 0.789, 0.875, and 0.701, respectively. The alpha (α), CR, and AVE values of behavioral intentions are reported as 0.752, 0.858, and 0.668. Perceived benefits are 0.809, 0.867 and 0.568. In addition perceived ease of use-values are 0.838, 0.885, and 0.606.; perceived enjoyment values are 0.756, 0.845, and 0.577. Lastly, the three items of social influence show Cronbach α, CR, and AVE values of 0.757, 0.861, and 0.673, respectively. Furthermore, all the hypotheses (except for the moderator payment mode) show that their discriminant validity (Table 3) meets the requirement suggested by Fornell and Larcker (1981); that is, the square root of each construct's AVE should be higher than its correlation with the remaining constructs.

**Table 2:** Convergent validity of measurement model.

**Constructs**	**Items**	**Loading**	**α**	**CR**	**AVE**
Actual behavior (AB)	AB1 AB2 AB3	0.836 0.854 0.821	0.789	0.875	0.701
Behavioral intention (BI)	BI 1 BI 2 BI 3	0.816 0.807 0.829	0.752	0.858	0.668
Perceived benefits (PB)	PB1 PB2 PB3 PB5 PB8	0.819 0.769 0.740 0.709 0.725	0.809	0.867	0.568
Perceived ease of use (PEOU)	PEOU1 PEOU2 PEOU3 PEOU4 PEOU5	0.791 0.784 0.783 0.801 0.732	0.838	0.885	0.606
Perceived enjoyment (PE)	PE1 PE2 PE3 PE4	0.782 0.793 0.749 0.711	0.756	0.845	0.577
Social influence (SI)	SI2 SI3 SI5	0.816 0.852 0.793	0.757	0.861	0.673

*AB, actual behavior; BI, behavioral intention; PB, perceived benefits; PEOU, perceived ease of use; PE, perceived enjoyment; SI, social influence*.

**Table 3:** Measurement model and discriminant validity.

**Measures**	**AB**	**BI**	**PB**	**PEOU**	**PE**	**SI**
Actual behavior	**0.837**					
Behavioral intention	0.722	**0.817**				
Perceived benefits	0.511	0.620	**0.753**			
Perceived ease of use	0.554	0.617	0.718	**0.779**		
Perceived enjoyment	0.539	0.579	0.509	0.595	**0.760**	
Social influence	0.619	0.538	0.321	0.389	0.446	**0.821**

*AB, actual behavior; BI, behavioral intention; PB, perceived benefits; PEOU, perceived ease of use; PE, perceived enjoyment; SI, social influence*.

^*^Boldface numbers are the squirt of the AVE.

### Structural Model

**Figure 2:**
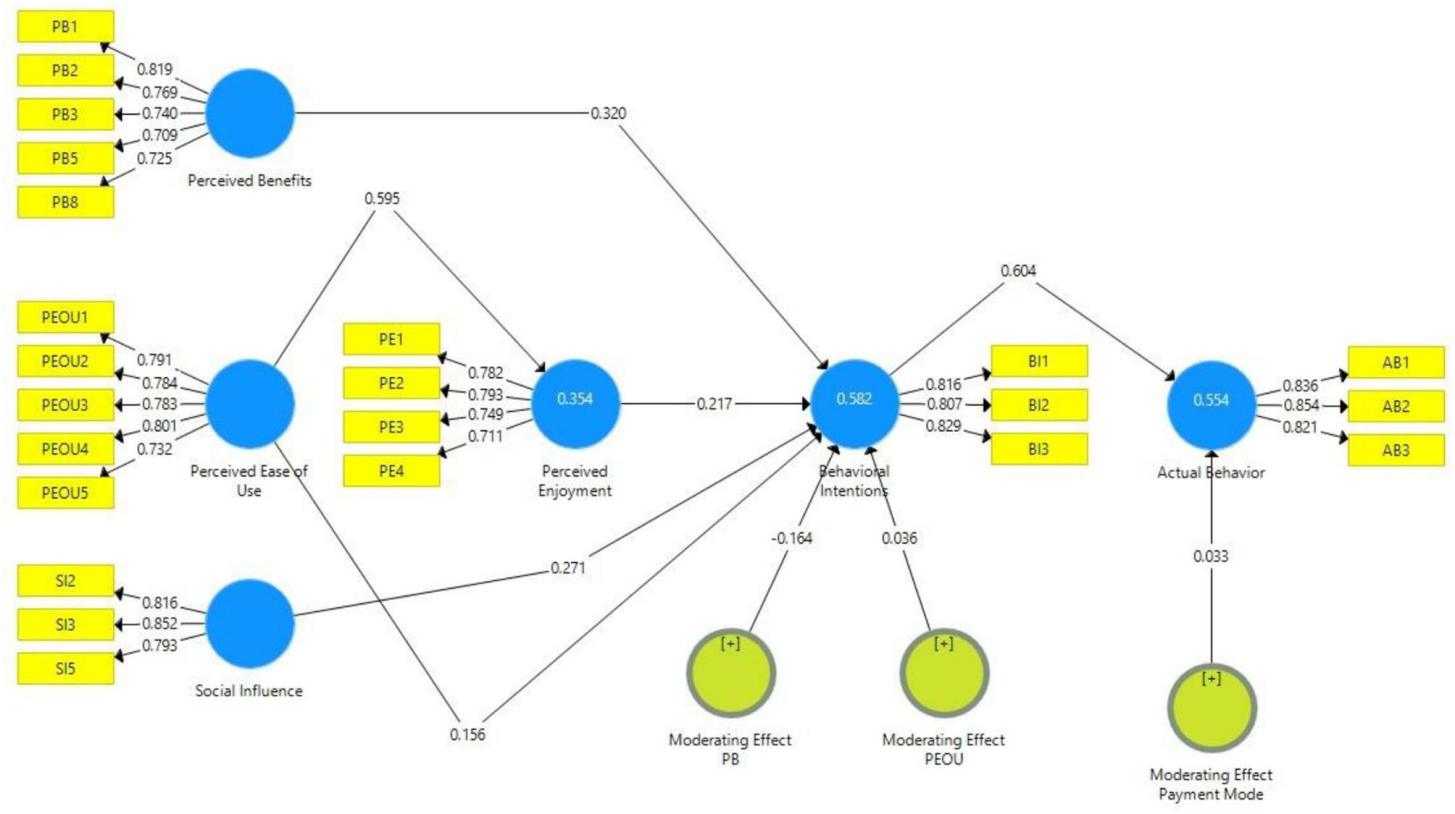
Structural model.

To assess the results, the estimated path coefficient of the structural model is analyzed. The results of variables and constructs are shown in Figure 2. The analysis shows that there is a positive and significant impact of perceived behavior, perceived ease of use, perceived enjoyment, and social influence on behavioral intention for online shopping, as their values are H_1_: β = 0.32, *p* < 0; H_4_: β = 0.156, *p* < 0.001; H_5_: β = 0.217, *p* < 0.001; H_7_: β = 0.217, *p* < 0.001, respectively. However, perceived ease of use also shows a significant and positive impact on perceived enjoyment given that H_6_: β = 0.595, *p* < 0.001.

PLS has been used to test moderating and mediating impacts, and special consideration has been given to assess relevant effects in a single model; PLS made it more sophisticated and allowed not to follow a causal step approach to evaluate mediating and moderating effects, whereas considering mediating and moderating effects with PLS is straightforward, and the outcomes give deep insights into advanced mediation and moderation analyses more accurately (Chin, 2010; Streukens et al., 2010; Nitzl et al., 2016). Hence, relevant effects have been assessed overall in a single model. To discuss the moderating roles of the model, it is evident from the results that gender shows significant moderation in perceived behavior and behavioral intention relationship: H_2_: β = −0.164, *p* = 0.006. In contrast, there is an insignificant moderation impact of gender on perceived ease of use and behavioral intention relationship: H_3_: β = 0.036, *p* = 0.501.

In addition, the moderation impact of payment mode also shows insignificance on behavioral intention and actual behavior relationship: H_8_: β = 0.033, *p* = 0.247). Lastly, the model has demonstrated a significant and positive impact of behavioral intention on the actual buying behavior of online consumers: H_9_: β = 0.604, *p* < 0.001). Therefore, H_1_, H_2_, H_4_, H_5_, H_6_, H_7_, and H_9_ are supported, whereas H_3_ and H_8_ are rejected based on the results. Detailed findings are shown in Table 4.

**Table 4:** Structural model results (hypothesis testing).

**Hypothesis**	**Relationship**	**β**	**Std Dev**	***t*-value**	**Sig Value**	**Decision**	**Q^**2**^**	**R^**2**^**
H_1_	PB → BI	0.320^***^	0.065	4.915	*p* < 0.001	Supported	0.371	0.554
H_2_	GENDER on PB → BI	−0.164^*^	0.060	2.742	*p* < 0.05	Supported		
H_3_	GENDER on PEOU → BI	0.036 ns	0.054	0.673	*p* > 0.05	Not supported		
H_4_	PEOU → BI	0.156^*^	0.065	2.404	*p* < 0.05	Supported		
H_5_	PE → BI	0.217^***^	0.058	3.753	*p* < 0.001	Supported		
H_6_	PEOU → PE	0.595^***^	0.042	14.138	*p* < 0.001	Supported	0.200	
H_7_	SI → BI	0.271^***^	0.053	5.080	*P* < 0.001	Supported		
H_8_	PM on BI → AB	0.033 ns	0.028	1.160	*p* > 0.05	Not supported		
H_9_	BI → AB	0.604^***^	0.055	10.980	*p* < 0.001	Supported	0.376	

* *p < 0.05*,

^**^p < 0.01,

*** *p < 0.05, and ns = not significant*.

*AB, actual behavior; BI, behavioral intention; PB, perceived benefits; PM, payment mode; PEOU, perceived ease of use; PE, perceived enjoyment; and SI, social influence*.

According to the criteria, the value of R^2^ must be greater than 0.2, as proposed by Hair et al. (2016). The present study shows an acceptable value of R^2^, which is 0.554. Furthermore, the value of Q^2^ has also been examined using Stone-Geisser's blindfold technique; this technique can be used to examine function fitting and cross-validation. However, this procedure is stated as a sample reuse procedure by Mikalef et al. (2017). If the value of Q^2>^ 0, it implies that the model has a predictive relevance (Hair et al., 2012). The Analysis shows that the behavioral intention (Q^2^ =0.371), perceived enjoyment (Q^2^ = 0.2), and actual behavior (Q^2^ = 0.376) variables show reasonable predictive relevance, demonstrating that their values are above 0.

## Discussions and Implications

### Discussion

The recent COVID-19 pandemic has changed the landscape of business processes and how they used to function. Prolonged lockdowns resulted in responses to the pandemic causing closures of several companies. However, it brought a new wave of online shopping all over the global market. Interestingly, when businesses went bankrupt and started the closure of their processes, the online market thrived and expanded by over 30–50% (Financial Times, 2021). This shifted the relevance and significance of the research domain once more toward examining key components shaping one's intentions and behavior in a certain way. Thus, the present study seeks to investigate determinants impacting, moderating, and mediating consumers' online buying behavior during the COVID-19 pandemic. The findings suggest several contributions in consumer behavior, advertising, social media, digitalized marketing, academia, and practical aspects of consumers' intention and behavior.

### Theoretical Implications

The present study has examined and concluded the determinants impacting the way consumers' online behavior has changed during COVID-19 in the context of Pakistan. For this purpose, the study has developed an integrated model based on the foundation of the well-established Technology Acceptance Model and Theory of planned behavior. First, the results of this research have validated the established scales of measuring consumers' online behavior in South Asian countries, specifically Pakistan. Second, the significant impact of perceived benefits, perceived enjoyment, ease of use, and social influence shows the generalizability and predictive power of TAM and TPB to measure consumers' behavior during the COVID-19 pandemic. This contributes to the academia and research and development in the stated domain so that further research could be carried out with the inclusion of constructs obtained from the technological model (TAM), psychological, and behavioral model (TPB) along with the other notion of social influence on consumers' behavioral intention leading to shaping ones' actual technology usage behavior. The study holds novelty as the context is different from that of routine consumers' online behavior; this implies insights into how consumers' intentions have changed drastically to opt for online buying. Interestingly, according to pre-COVID times, some consumers showed reluctance to go for online buying considering facilitating conditions, i.e., payment mode (Triandis, 1977, 1980; Pang et al., 2016). However, during the COVID-19 pandemic, the same broader consumer base shifted drastically to opt for online buying. The study reveals a new research realm to extend relevant theoretical paradigms to examine the impact of the external environment on consumers' buying intention and behavior.

Second, the integrated model with the role of mediation and moderation implies that theory predicts consumers' intention across situations; the present study has shown its generalizability during the time of a pandemic. This paves the way for further theoretical contribution in “crisis times” by introducing key determinants in cross-cultural and longitudinal analyses.

### Practical Implications

Based on the results of this study, the following practical implications have been proposed:

First, the study provides supporting evidence of perceived benefits sought by consumers when buying online. It implies that when a consumer enjoys buying online, it influences their intention to choose purchase behavior in the long run. As consumers find it convenient, businesses need to work on enhancing website design and logistics systems to make shopping more user-friendly and prompt. Interactive and appealing website designs will make one's online experience enjoyable by providing superior images and photos of products/services, proper availability of product/service descriptions, and previous reviews on the same or related products. On the contrary, a complicated website and delays in distribution and logistics will obliterate the purpose of the “convenience” sought by consumers.

Second, perceived ease of use has shown a significant positive impact on perceived enjoyment and intention, indicating that perceived enjoyment mediates the effect of perceived ease of use on behavioral intention. It reveals that a consumer enjoys more when there is more ease for them to use technology. Hence, it stimulates one's intention toward online buying. Therefore, businesses need to work on their online service portals, availability of mobile phone website options, online check-out counters, guest check-out counter chatbots, and advanced navigation options from one product to another to make it effortless and user-friendly to enhance consumers' shopping experience.

Third, the role of gender has been studied widely to understand how gender as a moderator plays its role specifically while managing or using tech-oriented systems (Venkatesh et al., 2003; Yousafzai and Yani-de-Soriano, 2012). In the present study, in Pakistan, it is evident from the results that gender plays a significant role in the relationship between perceived benefits and behavioral intention, whereas it has an insignificant role in the relationship between perceived ease of use and behavioral intention. For the moderating role of gender in the relationship between perceived benefits and behavioral intention relationship, the coefficient is negatively significant, which means that although the relationship shows significance, it is weaker in nature.

The insignificant and weak moderating role of gender in the relationship between perceived ease of use and behavioral intention, and in that between perceived benefits and behavioral intention, reveals that the studied relationships are not affected by the gender of a consumer. One's perceived ease of use and perceived benefits may tend toward forming online intention regardless of what gender the person belongs to. Businesses must employ strategies considering gender-neutral online portals, website designs, and online shopping services regarding technology's ease of use and perceived benefits.

In addition, findings of payment mode have shown an interesting insight that payment mode has no impact on consumers' online buying. According to previous studies, the trust factor related to online privacy had been a vital issue, and consumers did not want to shop online because of fraudulent cases, specifically in developing countries. However, the present study reveals that COVID-19 circumstances left consumers with no choice but to adhere. This shift brought a considerable consumer market to the e-commerce sector, which was first-time online buyers (Statista, 2022). When consumers start trusting online payment structures in Pakistan, businesses need to make sure they take payment security as a priority, develop transaction systems, and secure their electronic payments *via* enhanced “secure electronic transaction” (SET) protocol. Lastly, social influence has also shown a key finding to positively impact buying intention. Businesses and marketers need to utilize such behaviors by involving more powerful bloggers/influencers who are famous among the public. These influencers may use social-friendly content in effortless ways to buy online. In this way, businesses may move from a more traditional way to a more personal level with their consumers. This may also include the creation of personal blogs, putting up fewer formal posts on social media handles, and decreasing the gap between brands and consumers by going through the personalization process.

### Limitations and Future Study

There is a significant scope to examine and investigate the external factors that impact consumers' shift toward online buying, specifically during crises like pandemics. Data have been collected from educated online users who are tech-savvy. However, education level and how users are learning technologies are constantly changing. Future research in this domain may be conducted with larger populations regardless of their educational status. Additionally, payment mode was one of the external factors used as a moderator to investigate its impact on online buying behavior; future research may include longitudinal studies to see if consumers' behavior persists across situations for payment mode or changes with difficult times like the COVID-19 pandemic. It will be significant to investigate diverse external factors that moderate one's intention-behavior relationship in particular times and changes over the period.

## Data Availability Statement

The raw data supporting the conclusions of this article will be made available by the authors, without undue reservation.

## Ethics Statement

Ethical review and approval was not required for the study on human participants in accordance with the local legislation and institutional requirements. Written informed consent from the participants was not required to participate in this study in accordance with the national legislation and the institutional requirements.

## Author Contributions

All authors listed have made a substantial, direct, and intellectual contribution to the work and approved it for publication.

## Conflict of Interest

The authors declare that the research was conducted in the absence of any commercial or financial relationships that could be construed as a potential conflict of interest.

## Publisher's Note

All claims expressed in this article are solely those of the authors and do not necessarily represent those of their affiliated organizations, or those of the publisher, the editors and the reviewers. Any product that may be evaluated in this article, or claim that may be made by its manufacturer, is not guaranteed or endorsed by the publisher.
